# Inhibitory Top-Down Control Deficits in Schizophrenia With Auditory Verbal Hallucinations: A Go/NoGo Task

**DOI:** 10.3389/fpsyt.2021.544746

**Published:** 2021-06-04

**Authors:** Qiaoling Sun, Yehua Fang, Yongyan Shi, Lifeng Wang, Xuemei Peng, Liwen Tan

**Affiliations:** ^1^Department of Psychiatry, Mental Health Institute of the Second Xiangya Hospital, Central South University, Changsha, China; ^2^Department of Clinical Psychology, Zhuzhou Central Hospital, Zhuzhou, China; ^3^Department of Clinical Psychology, The Third Xiangya Hospital of Central South University, Changsha, China; ^4^Department Psychology, Xiangtan Central Hospital, Xiangtan, China

**Keywords:** P3, event-related potential, schizophrenia, auditory verbal hallucination, inhibitory top-down control

## Abstract

**Objective:** Auditory verbal hallucinations (AVH), with unclear mechanisms, cause extreme distresses to schizophrenia patients. Deficits of inhibitory top-down control may be linked to AVH. Therefore, in this study, we focused on inhibitory top-down control in schizophrenia patients with AVH.

**Method:** The present study recruited 40 schizophrenia patients, including 20 AVH patients and 20 non-AVH patients, and 23 healthy controls. We employed event-related potentials to investigate the N2 and P3 amplitude and latency differences among these participants during a Go/NoGo task.

**Results:** Relative to healthy controls, the two patient groups observed longer reaction time (RT) and reduced accuracy. The two patient groups had smaller NoGo P3 amplitude than the healthy controls, and the AVH patients showed smaller NoGo P3 amplitude than the non-AVH patients. In all the groups, the parietal area showed smaller NoGo P3 than frontal and central areas. However, no significant difference was found in N2 and Go P3 amplitude between the three groups.

**Conclusions:** AVH patients might have worse inhibitory top-down control, which might be involved in the occurrence of AVH. Hopefully, our results could enhance understanding of the pathology of AVH.

## Introduction

Auditory verbal hallucinations (AVH) are vivid perceptions of sound that occur without corresponding external stimuli and have a strong sense of reality. It occurs in 60–80% of schizophrenia patients (1) and causes multiple dysfunctions and poor control of behaviors (2, 3). Schizophrenia patients with AVH may have an increased tendency toward violent behaviors or acts (4–6), which may pose a threat and serious burden to society and their families.

Controlling and eliminating symptoms of hearing voices is difficult in treatment. Many efforts have been devoted to the treatment of auditory hallucinations, but the results still remain unsatisfactory (7). Studies have been conducted to investigate the efficacy of antipsychotic medications for AVH in schizophrenia patients, which exhibited a significant treatment effect of several typical and atypical antipsychotics (8, 9). However, there are still a considerable minority of schizophrenia patients showing no treatment effect of antipsychotics (9) and AVH can be drug-resistant and become chronic in around 25% of schizophrenia patients (10). Brain stimulation and psychological intervention are also applied in the treatment for AVH, but the curative effect is not ideal. For example, transcranial magnetic stimulation may reduce the frequency and severity of AVH, but the efficacy effect size of 1 Hz transcranial magnetic stimulation was just 0.44, supported by meta-analysis (11). And cognitive-behavioral therapy, which is considered as the most investigated psychological intervention of AVH, has an average effect size of 0.44 (12).

Schizophrenia patients experiencing AVH usually report that they have been hearing words, sentences and conversations which often comment on their thoughts. Healthy individuals who experience this are typically aware that the “voices” they hear are false perceptions and originate from their mind. In addition, they seem to be able to cope with this false perceptual experience by recruiting inhibitory control functions. However, hallucinating schizophrenia patients seem to focus on “voices” and appear less able to exhibit inhibitory control and thus are less able to attend to events around them (13). It is suggested that inhibitory top-down control functions should play an essential role in modulating experience of voice that originates from one's mind or an external source. In other words, deficits in inhibitory top-down control process may be important in AVH (14).

Neuroimaging studies have shown that AVH results from a variety of alterations in brain structure (15, 16). Findings of structural imaging studies converge on gray matter reductions in the superior temporal gyrus, insula and inferior frontal gyrus as well as abnormalities in the connecting white matter between these regions, which associated with the processing of auditory verbal stimuli and executive control functions. Paulik et al. conducted a study with 589 undergraduate students who were drawn into high- and low-predisposed groups using the Launay-Slade Hallucination Scale (LSHS). They found that compared with the low LSHS group, the high LSHS group showed significantly increased false alarms on critical “inhibitory” runs (17). And according to Waters et al. and Badcock et al., the damage level of inhibitory control ability was positively correlated with the auditory hallucination severity (18, 19).

Electrophysiological approaches also provide important insights into the underlying mechanisms of AVH. Especially the event-related potentials (ERP), which are real-time measures of neural activity with high temporal resolution and promising tools to explore brain dynamics that underlie deficits during task performance. ERP recordings in schizophrenia with AVH have shown deficits in a series of components, including the early, P50 (20) and mismatch negativity (21) and the later, P3. P3 is a measure of inhibitory control, which has been well-studied in schizophrenia, but only few studies have reported the relationship between P3 and AVH in schizophrenia (22). Top-down inhibitory control measured in ERP often via the dichotic listening test, and the study have found that more dysfunctional top-down inhibition seemed to mediate the association between impairment to affective theory of mind and severity of hallucinations (23). The Go/NoGo task is a classical paradigm, in which participants respond to the frequent “Go” stimuli as quickly as possible and avoid button pressing reaction in the infrequent “NoGo” stimuli (24, 25). N2 and P3 in the Go/NoGo task are closely related inhibitory control (26–28). Despite intensive investigations, the AVH remains a poorly understood feature of schizophrenia. Many studies found that schizophrenia patients showed deficits in inhibitory control (29–31), but most of these studies did not make further analysis with regard to symptoms. Only a few studies argued that patients experiencing no AVH may not have obvious deficits in inhibitory control (18). It is not clear whether the inhibitory control deficits in AVH patients stem from the disorder or the symptom. Additionally, results from Go/NoGo task may provide more support for inhibitory control deficits in AVH patients. Thus, in the present study, we aim to investigate inhibitory top-down control in schizophrenia patients with and without AVH using a Go/NoGo task.

## Materials and Methods

### Participants

The schizophrenia patients were recruited from the Outpatient Department of Psychiatry, the Second Xiangya Hospital of Central South University, China. The study and its aims were explained to all the participants and informed consent from them was obtained. This manuscript of the informed consent was obtained in compliance with the Helsinki Declaration. The inclusion criteria are (a) met ICD-10 criteria for schizophrenia; (b) aged between 18 and 30 years; (c) normal or corrected-to-normal vision; (d) right-handed; and (e) education level > 9 years and able to complete the test. The exclusion criteria are (a) with a history of head injury resulting in loss of consciousness; (b) alcohol or drug dependence; and (c) had taken an ERP test before.

Twenty patients with AVH were asigned to the AVH group and 20 patients who had never experienced AVH were assigned to the non-AVH group. All the patients were assessed by two senior clinical psychiatrists using the Positive and Negative Syndrome Scale (PANSS) (32). The healthy controls (*n* = 23) were recruited from the local community by advertisement. The demographic and clinical characteristics of all the subjects are demonstrated in Table 1.

**Table 1:** Demographic and clinical characteristics of AVH patients (*n* = 20), non-AVH patients (*n* = 20) and Healthy controls (*n* = 23).

**Characteristics**	**AVH patients**	**Non-AVH patients**	**Healthy controls**	**Statistics values**	***p***
Sex (male/female)	14/6	15/5	17/6	χ^2^ = 0.142	0.932
Age (years)	24.85 ± 5.57	25.10 ± 4.85	23.09 ± 3.01	1.278	0.286
Education (years)	13.00 ± 2.49	13.95 ± 2.82	15.22 ± 1.00	7.576	<0.001
Duration of illness (months)	25.45 ± 21.00	23.05 ± 25.76	-	0.323	0.749
PANSS-P3	4.45 ± 1.50	1.05 ± 0.22	-	10.000	<0.001
PANSS total score	61.55 ± 12.09	60.45 ± 18.26	-	0.224	0.824
PANSS positive score	18.05 ± 5.37	13.95 ± 3.95	-	2.749	0.009^*^
PANSS negative score	15.00 ± 6.52	16.95 ± 7.71	-	0.864	0.393
PANSS general psychopathology	28.50 ± 6.51	29.55 ± 9.26	-	0.415	0.681

*A hyphen “-” was used when the data were unavailable or there was no data.*

* *p < 0.05*.

### Stimuli and Task

Participants completed the Go/NoGo task in an electrically shielded, sound-attenuated room. Participants were positioned about 100 cm away from the screen. The Go/NoGo program was presented using the E-prime 2.0 software. The task begins with a non-informative cue (a small white cross) for 1,000 ms, and then the stimulus (K or X) was presented for 500 ms, followed by a blank screen for 500 ms. The “K” stimuli, as the Go stimulus, requires a button press response as quickly and accurately as possible, and its probability to appear is 2/3, with 240 times of appearance in total. The “X” stimuli, as the NoGo stimulus, requires non-response and its probability to appear is 1/3, with 120 times of appearance in total. The rare “X” stimuli are set to ensure that the NoGo reaction is the non-dominant response, and thus more attention is needed to carry it out correctly. Only correct Go responses (press within 200–1,000 ms after a Go-stimulus) and NoGo responses (no press after a Nogo stimulus) were recorded.

### Recording and Data Processing Procedures

Continuous electroencephalography (EEG) data were recorded using the BrainAmp MR (Brain Products, Germany). The electrode cap consists of 32 Ag/AgCl electrodes in accordance with a modified international 10 - 20 system. Vertical electro-oscillogram was recorded from one electrode fixed above the left eye, and the horizontal electro-oscillogram was recorded from one electrode fixed at the outer canthus of the right eye. The reference electrode was at FCz. All signals were digitalized with a sample rate of 500 Hz and with a frequency band from 0.1 to 100 Hz. Electrical impedance for each site was below 5 kΩ throughout the experiment. Offline data were processed with the Brain Vision Analyzer 2.0 system (Brain Products GmbH, Germany). EEG data were referenced to the average of mastoids (TP9 and TP10). EEG signals were bandpass filtered using a 0.5 to 30 Hz (50 Hz notch) rate. Eye movements and eye blinks were removed using an independent component analysis. Artifact rejection procedures were applied to all epochs (−200 ms pre-stimulus to 1,000 ms post-stimulus), with a baseline correction from −200 ms to 0 ms pre-stimulus. Epochs were averaged using only correct attempts according to the condition (Go, NoGo). The N2 and P3 amplitude were defined as the global maximum value to baseline at signal subject level (N2, 200–300 ms post-stimulus; P3, 300–500 ms post-stimulus).

### Statistical Analysis

Statistical analyses were completed using the SPSS19.0 software package (Statistics Product and Service Solutions), Chinese version. Sex differences between groups were analyzed using χ^2^ test. Differences in age between the three groups were assessed using one-way ANOVA. Education difference was evaluated using Welch's ANOVA, and Games-Howell was used for the *post-hoc* test. Clinical differences between two patient groups were evaluated using *t*-test. Amplitude and latency of N2 and P3 were evaluated with repeated-measures ANOVA, with group (AVH patients, Non-AVH patients, Healthy controls) as between-subject variable, and trial category (Go, NoGo) and topographical site [Frontal (Fz, F3, F4), Central (Cz, C3, C4), Parietal (P3, Pz, P4)] as within-subject variables. Age and education were unconcerned covariates. For behavioral data, one-way ANOVA was used to analyze the response time of the correct Go attempts. The accuracy (i.e., button presses in the Go trials and no responses in the NoGo trials) were investigated using a repeated-measures ANOVA with trial category × group. The threshold for statistical significance was set at *p* < 0.05. Statistical analyses were adjusted for variance non-sphericity using the Greenhouse-Geisser correction (33). All the *post-hoc* analyses were adjusted using the Bonferroni adjustment.

## Results

### Demographic Data

There were no significant differences (*p* > 0.5) in age and sex between all the groups but there was significant difference in the years of education (χ^2^ = 7.576, *p* < 0.001). Healthy controls had a higher education level than the AVH patients and non-AVH patients (*p* < 0.05). For the PANSS scale, the AVH patients had higher scores than non-AVH patients (*p* < 0.005) in only PANSS-P3 and PANSS positive symptoms. The two patient groups did not significantly differ in the duration of illness and the scores of other PANSS items.

### Behavioral Data

**Figure 1:**
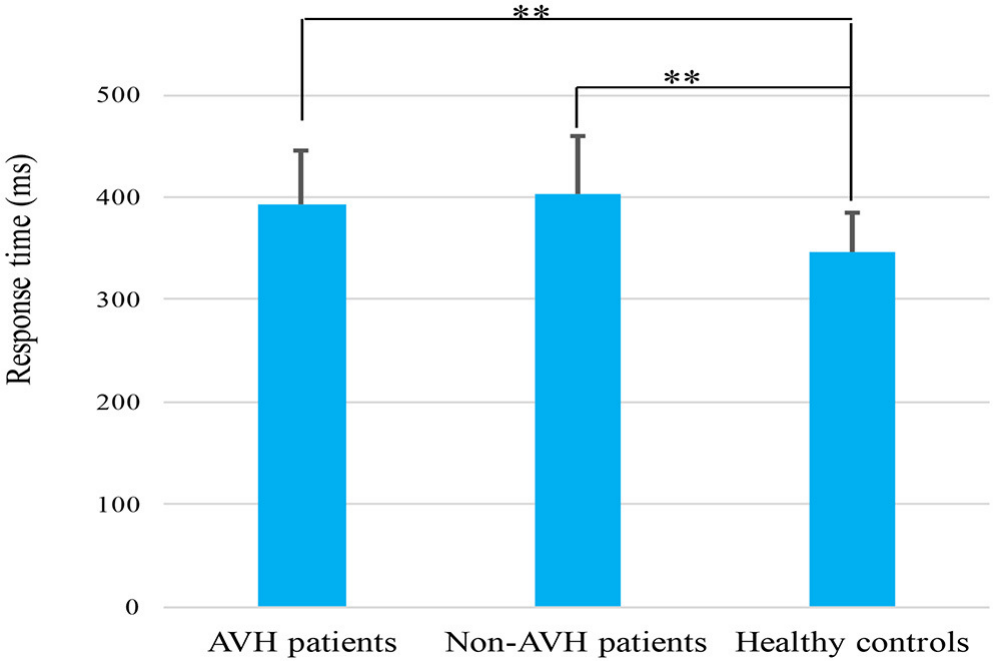
Mean response time elicited by correct Go trials in AVH patients, non-AVH patients and healthy controls. Error bars denote the standard error. **p < 0.01.

The three groups differed significantly in Go reaction time (RT) (*F* = 8.118, *p* = 0.001). The Go RT of the healthy controls was significantly shorter than those of the two patient groups (AVH patients, *p* = 0.018; Non-AVH patients, *p* = 0.005) (Figure 1). The main effect of trial category revealed that all the participants made more accurate responses in the Go trials than in the NoGo trials (*F* = 5.493, *p* = 0.022). A main group effect was found (*F* = 4.910, *p* = 0.011), and healthy controls had a higher accuracy than the AVH patients (*p* = 0.042) and non-AVH patients (*p* = 0.020). No significant trial category × group interaction was found (*F* = 0.604, *p* = 0.550).

### N2

**Figure 2:**
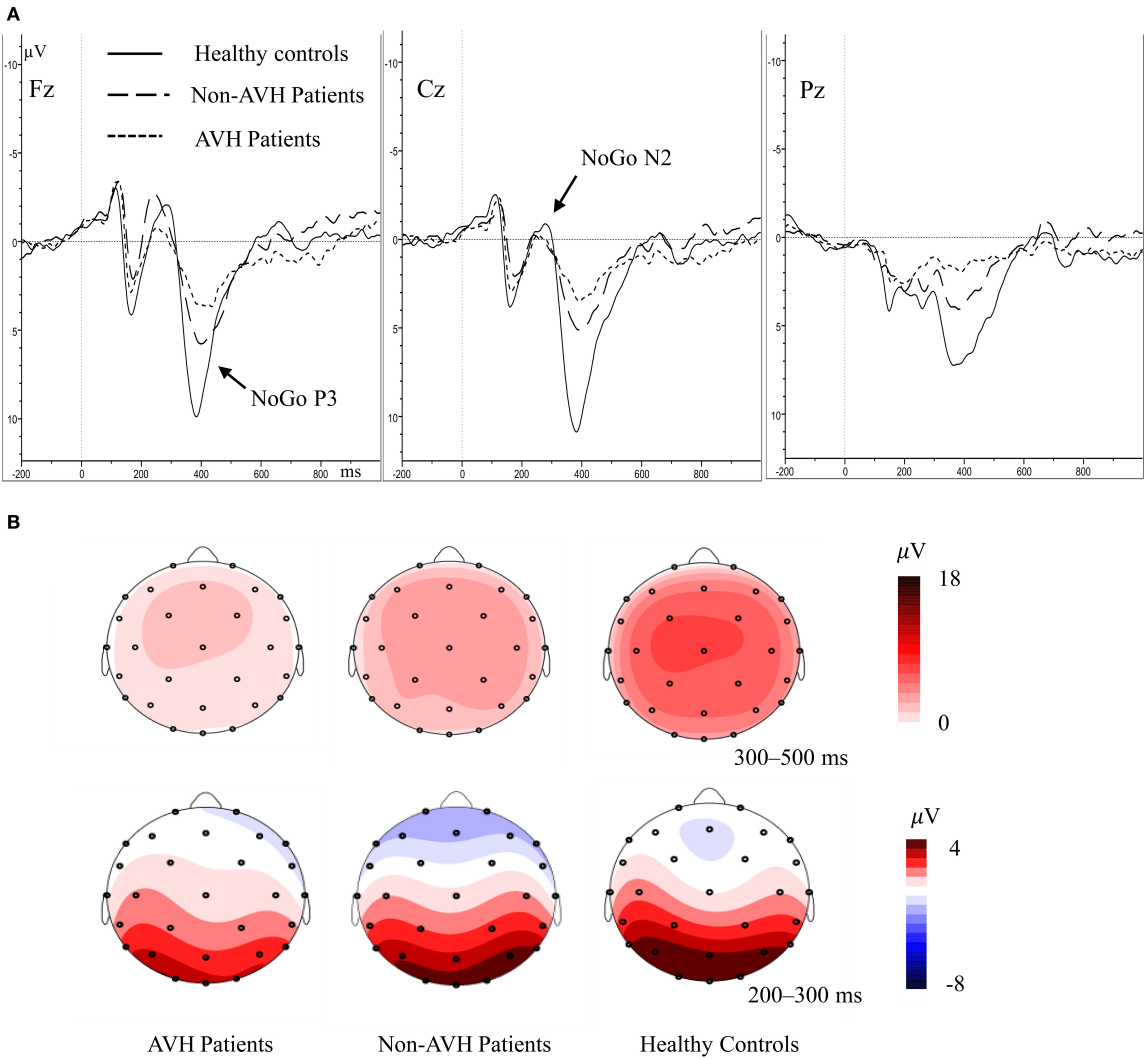
**(A)** Averaged waveforms for NoGo trials in AVH patients, non-AVH patients, and healthy controls. **(B)** Grand-averaged topographical maps for NoGo trials within 200–500 ms.

For N2 amplitude, no significant main effect of group was found (*F* = 0.269, *p* = 0.765). The main effect of trial category (*F* = 23.096, *p* < 0.001) and topographical site (*F* = 6.392, *p* = 0.002) were found; however, a significant interaction of trial category × topographical site (*F* = 12.534, *p* < 0.001) was also observed. *Post-hoc* tests demonstrated that the frontal (*p* < 0.001) and central (*p* < 0.001) areas has higher NoGo N2 amplitude than the parietal area (Figure 2). With regard to N2 latency, the main effect of group was not significant (*F* = 1.077, *p* = 0.347), indicating that the N2 latency was not different among three groups. A significant main effect of trial category was observed (*F* = 15.601, *p* < 0.001); however, a significant interaction of trial category × topographical site (*F* = 12.534, *p* < 0.001) was also observed. *Post-hoc* tests demonstrated that the NoGo N2 latency was longer, compared to Go N2 latency in the frontal and central areas.

### P3

**Figure 3:**
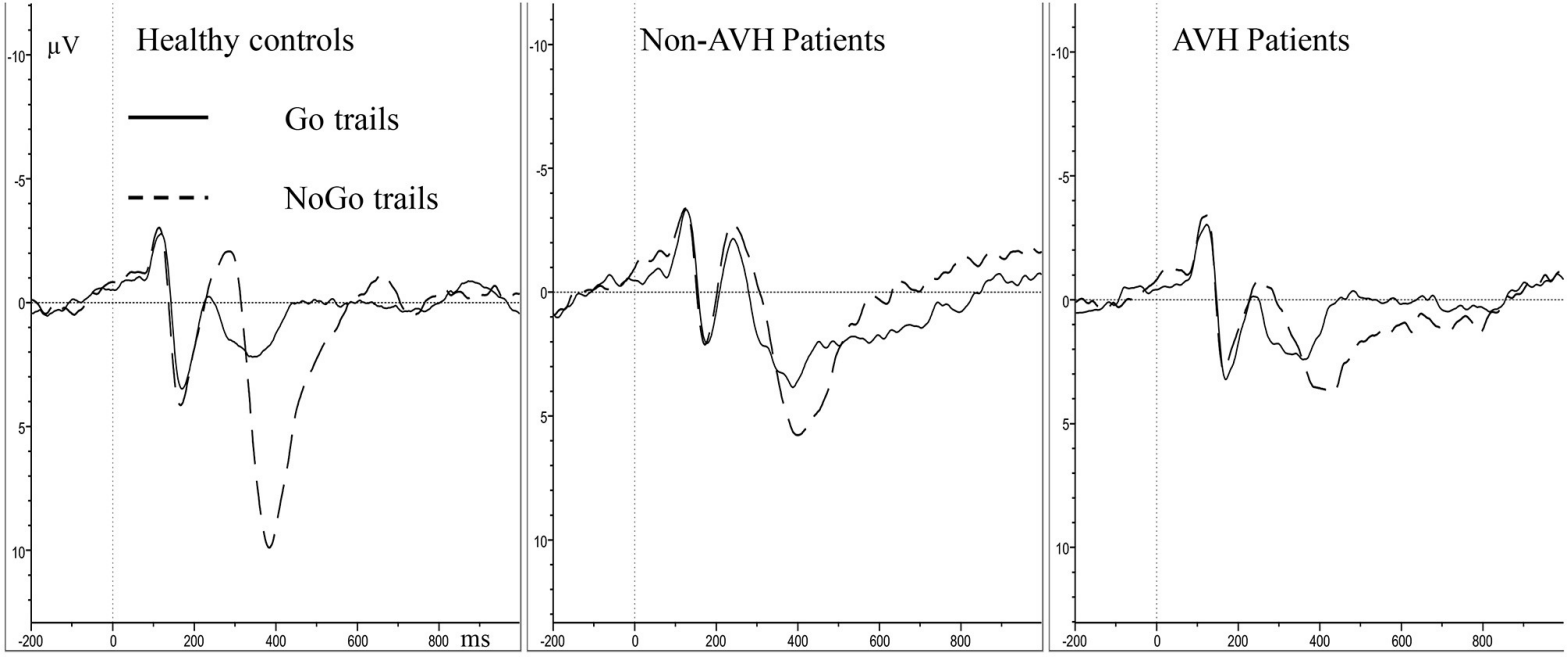
Averaged waveforms at Fz in Go and NoGo trails.

In P3 amplitude, the main effect of group (*F* = 10.419, *p* < 0.001), trial category (*F* = 38.215, *p* < 0.001) and topographical site (*F* = 9.303, *p* = 0.001) were observed separately, and a significant trial category × topographical site × group interaction (*F* = 4.530, *p* = 0.020) was also observed. Simple effect and *post-hoc* tests revealed that the healthy controls had higher NoGo P3 amplitude than the two patient groups, and the non-AVH patients had higher NoGo P3 amplitude than the AVH patients in all the topographical sites (Figure 2, frontal, *p* = 0.020; central, *p* = 0.046; parietal, *p* = 0.048); no significant difference was found of Go P3 amplitude between the three groups; NoGo P3 amplitude was larger than Go P3 amplitude (Figure 3), but this effect was not significant in the central brain region of the AVH patients (*p* = 0.865) and in the parietal area of the non-AVH patients (*p* = 0.544); and in all the three groups parietal showed smaller NoGo P3 than the frontal (AVH patients, *p* = 0.019; non-AVH patients, *p* = 0.008; healthy controls, *p* = 0.004) and central (AVH patients, *p* < 0.001; non-AVH patients, *p* < 0.001; healthy controls, *p* < 0.001) areas. No group main effect was found with regard to P3 latency. For detailed results of repeated-measures ANOVA of N2 and P3, see Supplementary Table.

## Discussion

In line with previous reports (29, 34), the present study also found that the two schizophrenia groups had longer Go RT and lower accuracy than those of the healthy controls, which might indicate that schizophrenia patients have inefficient cognitive processing. No significant difference was found in Go P3 amplitude between the three groups. The NoGo P3 amplitude in healthy controls was larger than the two patient groups, and the AVH patients had the smallest NoGo P3 amplitude, indicating that the inhibitory control was weakened in schizophrenia patients and inhibitory top-down deficits may also be related to AVH.

Inhibitory control is impaired in schizophrenia patients (30, 31). Patients with schizophrenia showed a significantly increased duration of the voluntary response inhibition process compared to healthy controls (35). A meta-analysis using the stop-signal task confirmed an inhibition deficit of moderate size in schizophrenia (36). Furthermore, schizophrenia patients showed a combination of a moderate deficit in response time with a moderate deficit in omission errors (30) in a meta-analysis of research using a go/no-go task, Conners' continuous performance task and sustained attention to response task. There were some circuit abnormalities underlying the response inhibition impairment in schizophrenia (37) and inhibitory control deficits were correlated with poorer prognosis in schizophrenia (38).

Compared to patients with minimal hallucinatory behavior and healthy controls, patients with pronounced hallucinations showed poorer inhibitory top-down control (23). Hugdahl et al., using a dichotic listening paradigm, found that the more frequent the hallucinations appear, the less the patients were able to use cognitive control in the forced-left instruction condition, indicating that patients experiencing AVH fail to use executive functions and cognitive control to avoid their engaging in the “voices” (39). Many neuropsychological studies have found that inhibitory control plays an important role in the AVH of schizophrenia patients (18, 40). Recent findings from neuroimaging studies have revealed that AVH in schizophrenia is associated with alterations in brain structure and function (41–43), which may provide the neural substrates for the production of AVH. Dysfunction in these neural substrates may produce internal auditory signals, and then patients with deficits in top-down control fails to suppress such information, which contributes to the failure to control the frequency and onset of these auditory signals effectively. These results suggest that impaired top-down control is involved in the formation of hallucinations (14, 44).

NoGo N2 may reflect the confirmation and preparation stage of inhibitory control, whereas NoGo P3 may correspond to the execution stage (27, 28). The present study found that, compared with healthy controls and Non-AVH patients, ERP abnormalities of AVH patients only appear in P3, which prompts that the neural mechanism of AVH may be related to the late inhibitory control process. The present study found no obvious inter-group difference in N2 latency, P3 latency and N2 amplitude between the AVH patients and healthy controls. RT is substantially prolonged in the AVH patients, but the latency of the P3 component is not, which may also suggest that the RT deficits arise from impairments in a late inhibitory control process (45).

In all the groups, the parietal area showed smaller NoGo P3 amplitude than the frontal and central area, prompting that NoGo P3 was mainly distributed in the frontal-central region, which was consisted with previous studies (29, 46). These findings suggest that AVH in schizophrenia patients may be associated with neuropathological abnormalities in frontal-central brain regions. Compared with non-AVH patients, AVH patients showed larger frontal gray matter volume (47) and decreased connection from the left inferior frontal gyrus to the left middle temporal gyrus (48). An abnormal structural network, including medial/inferior frontal areas, may reflect a neural signature of AVH in the expression of specific characteristics of AVH (49).

The results suggest us that it is possible to develop cognitive remediation that target top-down processing for AVH in schizophrenia patients. Basic neuroscience research has elucidated that the behavioral and biological determinants of neurophysiological change mediated by alterations in synaptic connection and neural network function (which termed “neural plasticity”). Additionally studies (50, 51) have shown that neural plasticity in adults requires intensive practice. Cognitive remediation refers to a series of treatments aimed to enhancing neurocognitive abilities, which can be carried out in a “bottom-up,” a “top-down” and a non-targeted training perspective (52). Training uses a “top-down” approach can train higher level cognitive processes, and this approach has been combined within broader training environments to simultaneously target both basic perceptual abilities, and higher level executive functions. The study use a computer-based training to enhances verbal memory in schizophrenia via a top-down and bottom-up approach have achieved good results (53). However, it still needs a lot of research to apply the top-down cognitive remediation to AVH in schizophrenia. Because the top-down mechanisms involved in AVH includes not only inhibitory control, but also other demains such as attention, prior knowledge/experience and emotional processes.

The present study has some limitations. Firstly, the sample size was relatively small, and the participants were predominantly male. Secondly, at the stage of research design, we set the education level > 9 years, but unfortunately, we failed to match the education years among the three groups. Although we did covariate analysis, but there is no guarantee that the effect of education level during this population is linear. So the mismatched education may increase the false positive rate of the results. Thirdly, a clinical interview, instead of a structured interview was used in the diagnosis of mental disorders. Hence, the accuracy of the diagnosis might not be optimal. In addition, part of our patients was being prescribed atypical antipsychotic medications. Whether the medicines influenced performance on these tasks is unclear. Therefore, we cannot rule out a possible normalizing effect of medicines and studies of unmedicated patients are required to clarify this.

The present study shows that inhibitory control was impaired in schizophrenia patients. AVH in schizophrenia may also be related to deficits in late inhibitory control process and neuropathological abnormalities in frontal-central brain regions. Our study provided some evidence that inhibitory top-down control may be involved in the occurrence of AVH.

## Data Availability Statement

The original contributions presented in the study are included in the article/Supplementary Material, further inquiries can be directed to the corresponding author/s.

## Ethics Statement

The studies involving human participants were reviewed and approved by Biomedical Ethics Board of the Second Xiangya Hospital. The patients/participants provided their written informed consent to participate in this study.

## Author Contributions

LT, QS, and XP conceived and designed the experiments. QS, XP, YS, YF, LW, and LT performed the experiments. QS and XP analyzed the data and drafted the manuscript. XP and YF made great contribution to the revision of the manuscript. All authors gave final approvement of the version to be published.

## Conflict of Interest

The authors declare that the research was conducted in the absence of any commercial or financial relationships that could be construed as a potential conflict of interest.
