# Supplementary material for: Inhibitory Top-Down Control Deficits in Schizophrenia With Auditory Verbal Hallucinations: A Go/NoGo Task
Source: Front Psychiatry. 2021 Jun 4;12:544746. doi: 10.3389/fpsyt.2021.544746 (PMC8211872; doi:10.3389/fpsyt.2021.544746)
Supplement: Supplementary file 1 [file Table_1.DOCX]

Supplementary table.

Repeated-measures ANOVA results of N2 and P3.

|  | | F | P | Partial  Eta-Squared |
| --- | --- | --- | --- | --- |
| P3 Amplitude | |  |  |  |
|  | trial category | 38.215 | < 0.001* | 0.389 |
|  | group | 10.419 | < 0.001* | 0.258 |
|  | topographical site | 9.303 | 0.001* | 0.134 |
|  | trial category $\times$ group | 17.213 | < 0.001* | 0.365 |
|  | trial category $\times$ topographical site | 76.360 | < 0.001* | 0.560 |
|  | group $\times$ topographical site | 1.452 | 0.233 | 0.046 |
|  | group $\times$ topographical site $\times$ trial category | 4.530 | 0.020* | 0.100 |
| P3 latency | |  |  |  |
|  | trial category | 13.104 | 0.001* | 0.179 |
|  | group | 1.868 | 0.163 | 0.059 |
|  | topographical site | 7.427 | 0.002* | 0.110 |
|  | trial category $\times$ group | 0.604 | 0.550 | 0.020 |
|  | trial category $\times$ topographical site | 3.930 | 0.025* | 0.061 |
|  | group $\times$ topographical site | 0.783 | 0.515 | 0.025 |
|  | group $\times$ topographical site $\times$ trial category | 0.637 | 0.624 | 0.021 |
| N2 Amplitude | |  |  |  |
|  | trial category | 23.096 | < 0.001* | 0.212 |
|  | group | 0.269 | 0.765 | 0.009 |
|  | topographical site | 6.392 | 0.002* | 0.596 |
|  | trial category $\times$ group | 0.267 | 0.767 | 0.038 |
|  | trial category $\times$ topographical site | 92.043 | < 0.001* | 0.085 |
|  | group $\times$ topographical site | 1.491 | 0.222 | 0.042 |
|  | group $\times$ topographical site $\times$ trial category | 1.100 | 0.349 | 0.040 |
| N2 latency | |  |  |  |
|  | trial category | 15.601 | < 0.001* | 0.369 |
|  | group | 1.077 | 0.347 | 0.035 |
|  | topographical site | 14.828 | < 0.001* | 0.027 |
|  | trial category $\times$ group | 1.460 | 0.240 | 0.050 |
|  | trial category $\times$ topographical site | 12.534 | < 0.001* | 0.040 |
|  | group $\times$ topographical site | 0.119 | 0.953 | 0.051 |
|  | group $\times$ topographical site $\times$ trial category | 2.642 | 0.050 | 0.022 |

*p < 0.05. Age and education were covariates.
